# Biosafety and biosecurity capacity building: insights from implementation of the NUITM-KEMRI biosafety training model

**DOI:** 10.1186/s41182-018-0108-7

**Published:** 2018-08-08

**Authors:** Betty Muriithi, Martin Bundi, Amina Galata, Gabriel Miringu, Ernest Wandera, Cyrus Kathiiko, Erick Odoyo, Martha Kamemba, Evans Amukoye, Sora Huqa, Mohammad Shah, Shingo Inoue, Yoshio Ichinose

**Affiliations:** 1 Nagasaki University Institute of Tropical Medicine Kenya Medical Research Institute Project, Nairobi, Kenya; 2 National Biosafety Authority, Nairobi, Kenya; 3 Kenya Medical Research Institute Project, Centre for Respiratory Diseases Research, Nairobi, Kenya; 4 Centre for Microbiology Research, Kenya Medical Research Institute Project, Nairobi, Kenya; 5 Centre for Infectious Disease Research in Asia and Africa, Nagasaki University Institute of Tropical Medicine, Nagasaki, Japan; 6 Department of Virology, Nagasaki University Institute of Tropical Medicine, Nagasaki, Japan; 7 NUITM-KEMRI c/o KEMRI-CMR, P.O. Box 19993-002, Nairobi, Kenya

**Keywords:** Biosafety, Biosecurity, Training, BSL-3 laboratory

## Abstract

The NUITM-KEMRI biosafety training program was developed for capacity building of new biosafety level three (BSL-3) laboratory users. The training program comprehensively covers biosafety and biosecurity theory and practice. Its training curriculum is based on the WHO biosafety guidelines, local biosafety standards, and ongoing biosafety level three research activities in the facility, also taking into consideration the emerging public health issues. The program’s training approach enhances the participant’s biosafety and biosecurity knowledge and builds their skills through the hands-on practice sessions and mentorship training. Subsequently, the trainees are able to integrate acquired knowledge and good practices into their routine laboratory procedures. This article describes implementation of the NUITM-KEMRI biosafety training program.

## Introduction

The NUITM-KEMRI biosafety training program was developed with a primary goal of training new biosafety level three (BSL-3) laboratory users before they begin working in the laboratory and to generally strengthen biosafety and biosecurity in research and diagnostic settings [1]. Containment laboratories are complex working environments, with a substantial potential of exposure of personnel and the public to infectious pathogens. Minimization of biosafety and biosecurity breaches should therefore be at the core of operation of containment facilities, although such environments cannot be entirely free of risks. The World Health Organization (WHO) manual [2] provides guidelines that enhance safety and security in containment laboratories if strictly adhered to. Further, among the provisions in the area of shared responsibility in biosafety and biosecurity is a provision for education and training that other internationally mandated regulatory regimes such as the Laboratory Biorisk Management Standards CWA 15793: 2011 revised in 2012 provides a basis for. However, despite the international commitment, compliance with training requirements in diagnostic and research settings is relatively poor while a standardized approach to training is still lacking. The NUITM-KEMRI BSL-3 biosafety training program was initiated in 2007 following installation of the BSL-3 laboratory [3, 4] and has been implemented annually to date. It equips BSL-3 laboratory workers and potential users with comprehensive biosafety and biosecurity knowledge and skills, towards ensuring compliance with safe practices that still remains low in most biomedical laboratories [5].

Several pathogenic microorganisms are endemic in Kenya, coupled with frequent outbreaks of infectious diseases. Recurrent outbreaks of cholera have been reported since 2014. Epidemic cholera was reported in 2017 by the Ministry of Health to the WHO involving 3967 suspected cases with 76 deaths [6]. Outbreaks of dengue fever and chikungunya virus have also been reported especially in the Coastal Region. Although polio outbreak has not been reported for quite some time, there is a risk of importation from neighboring areas whose health care infrastructure is weak due to socio-political instability. Yellow fever and Rift Valley fever still pose a public health threat due to prevalence of competent vectors among other factors. Given the huge burden of infectious diseases, the potential of encountering unexpected infectious agents in routine samples is high. The frequent occurrence of these diseases caused by risk group two and three agents and their associated morbidity and mortality therefore justifies the need for capacity building of laboratory staff, particularly those working in BSL-3 laboratories and laboratories with enhancements over and above statutory level two standards. As Ritterson and Cassagrande [7] noted, human error is an important cause of laboratory accidents. Moreover, laboratory-acquired infections are still rampant despite firmly established good microbiological techniques in most laboratories.

Globally, the WHO laboratory biosafety guidelines, the CEN Workshop Agreement (CWA) 15793 Laboratory Biorisk Management International consensus document, and the Biological Weapons Convention (BWC) among other international instruments guide biosafety and biosecurity management. The CWA 15793 is intended to be used as a framework to implement national guidelines and policies related to biosafety and biosecurity. Locally, the Kenya biosafety policy guidelines in addition to the Occupational Safety and Health Act (OSHA) 2007 regulation, Environmental Management and Coordination (Amendment) Act 2015, and National Infection Prevention and Control Guidelines for health care services in Kenya offer guidelines in biosafety and biosecurity management in the country. These guidelines provide a basis for biosafety and biosecurity training.

BSL-3 biosafety training is mostly provided by research institutions, universities, and or regional biosafety associations, networks, or agencies [8, 9]. Training curriculum is developed based on the training needs of the target group among other determinants. Homer et al. [10] for instance proposes ten key training concepts for workers assigned to BSL-3 laboratories that cover a broad range of core biosafety and biosecurity concepts. On the contrary, Hartman, et al. [8] describes a facility-specific training program whose content covers operation of an institution’s BSL-3 laboratory, an approach also used by most universities that provide BSL-3 biosafety training. The WHO manual [2] and other internationally recognized documents are also used to develop training curriculums depending on institutional needs and settings [11]. Despite the need to integrate BSL-3 laboratory training with BSL-3 laboratory operations, availability of resources and human capacity, as well as lack of legal framework upon which training guidelines can be established, remain to be important impediments [12].

The NUITM-KEMRI biosafety training program was developed based on the WHO laboratory biosafety manual (2004), with input from ongoing research studies as well as the prevailing public health conditions. Training objectives were designed around the knowledge and skills that the trainees are expected to demonstrate after the training, while factoring in the latter conditions. Training content was therefore developed in consultation with experienced BSL-3 laboratory personnel, biosafety officers, and relevant scientists.

## Training procedure

### Training content

The training content is largely based on the WHO Laboratory Biosafety Manual [2]. The manual primarily addresses laboratory biosafety, providing guidelines for establishment of containment principles, and practices that prevent unintentional exposure to biological agents and toxins.

Table 1 below outlines the training program. An initial written quiz or survey is first administered. The quiz covers all the contents taught during the training while the survey is based on a single predetermined topic. Initial assessment is used to gauge the trainees’ baseline knowledge and awareness of biosafety and biosecurity principles. In the next phase, trainees are subjected to theoretical and practical training. Theoretical training covers concept and knowledge, the NUITM P3 laboratory, and operations inside the BSL-3 laboratory categories. The concept and knowledge category introduces trainees to the concept of biosafety and biosecurity. A rationale for biosafety and biosecurity is provided through a detailed description of laboratory-acquired infections through which the impact of breaching biosafety and biosecurity principles is discussed. Also covered under this category is risk group classification of microorganisms and biosafety classification of laboratories alongside risk assessment. These modules enable trainees to allocate necessary safety strategies to individual microorganisms based on potential risk borne. Biosafety containment at each biosafety level is discussed too including biosafety equipment, biosafety rules, regulations, and waste management at each level. Additionally, trainees learn basic mycology, virology, and bacteriology through application modules that provide practical examples on handling of select pathogens.

**Table 1:** Training program outline

Training phase	Description	Method
Initial assessment	Pre-training quiz Pre-training survey	Sit-in examination
Training phase	Theory • Concept and knowledge ◦ Background of laboratory-acquired infections ◦ Microorganisms risk group classification ◦ Biosafety containment levels ◦ Biosafety containment strategies; facilities and techniques ◦ Sample packaging and transportation ◦ Basic mycology, virology and bacteriology ◦ Biological and chemical waste management ◦ Biosecurity • NUITM BSL-3 laboratory: features, maintenance and management ◦ Laboratory design ◦ Physical and operational features of the NUITM P3 lab ◦ Biosafety equipment ◦ Routine care and maintenance • Operations inside the BSL-3 laboratory ◦ Laboratory entry checkpoints ◦ Documentation ◦ Decontamination Appropriate use of personal protective equipment ◦ Protocols and procedures for working in the BSL-3 laboratory ◦ Exit procedures and trouble Practicum ◦ Site visit to the checkpoints ◦ Demonstration of the correct procedure of entering, working inside the facility and exiting the BSL-3 laboratory ◦ Hands-on evaluation session	• Lectures • Discussion • Demonstration by trainers • Demonstration by trainees • Hands-on practice by trainees
Final assessment	Pre-training quiz Pre-training survey	

In the second category, the NUITM BSL-3 laboratory system is described. The physical and operational features of the laboratory and the role each plays in maintaining containment is first described. Biosafety equipment including personal protective equipment (PPE) are reviewed too, alongside routine care and maintenance of the facility. The last category covers the theory of operation of the BSL-3 laboratory, from entry into the laboratory to exit following completion of an experimental procedure. Entry usually starts with routine monitoring of checkpoints. Each checkpoint and required documentation is therefore described followed by protocols and procedures for working in the BSL-3 laboratory. Among the checkpoints are the cell culture room where availability of the BSL-3 suit is confirmed, the machine room for recording of manometer reading, and the ante room where BSL-3 suit operational parameters are observed and recorded. BSL-3 laboratory equipment including safety equipment are then described in detail as well as their operation, followed by a description of the systematic exit procedures.

The practical session is conducted based on the content covered in the second category. Demonstration and practice on donning and doffing of PPE is conducted followed by hands-on training on entering, working and leaving the BSL-3 laboratory. During this session, trainees are shown how to use the biosafety cabinets, autoclaves, and centrifuges. Sample handling is also covered, including retrieval, experimentation, disposal, storage, and inventory. The training phase is followed by the final assessment phase during when a written assessment similar the initial assessment is administered. Whenever applicable, a final topical survey is also administered. These are used to measure knowledge gained during the workshop.

### Training delivery

The training program is administered through an annual 3-day workshop. Theoretical training is conducted during the first day and part of the second day. Practical training begins after theoretical training on the second day and on the third day. Training evaluation follows practical training towards the end of the workshop. A discussion session is finally held, during which the written evaluations are discussed and questions arising from all sessions of the workshop responded to.

The training program is delivered through lectures, demonstrations, and hands-on training [1, 14]. Lectures are used to deliver theoretical content while demonstrations and hands-on training are used to administer the practical content.

### Training participants and trainers

Trainees are drawn from research centers and related research institutions. They are usually laboratory staff with experience in lower biosafety level laboratories, staff with access to the BSL-3 laboratory, or potential BSL-3 laboratory users. Trainees are mostly virologists and bacteriologists. Prerequisite requirement for inclusion in the workshop is knowledge and experience in basic microbiology practices. At least 12 trainees are recruited every year.

Trainers are sourced internally and externally. Trainers of introductory and practical modules are usually previously trained BSL-3 laboratory staff with at least 3 years of experience in BSL-3 settings, frequent users of the BSL-3 laboratory, with an academic background of medical laboratory sciences. Application modules are administered by research officers. These are majorly investigators conducting studies on an organism of biosafety interest. They must be familiar with good microbiology practices. Biosafety and biosecurity professionals are engaged where applicable.

### Training feedback

Feedback on training is obtained to examine the level of satisfaction of the trainees. Structured questionnaires designed to collect trainees’ opinions on training content, quality of the training materials, suitability of training approaches, and overall impression of the entire workshop are used. Questionnaires are anonymous and consist of yes and no, and open ended questions, for each module and the overall training workshop.

Resulting information is used for continuous improvement of the training program. For instance, feedback on the duration of training yields opinions on the adequacy of the allocated time while questions addressing training content yield feedback on curriculum coverage and the suitability of the training materials used.

### Training evaluation

Training is evaluated using written evaluations. The post-training test is issued after the training while the hands-on training test is administered after the hands-on training session. A total of 38 multiple-choice questions are administered. Whenever applicable, topical pre- and post-training surveys are conducted, or a pre-training test similar to the post-training test administered.

Pre-training scores are normally lower than post-training scores. A significant improvement is however observed in the post-training evaluation in most cases. Medina et al. [15] reported a statistically significant increase in the mean score by 30% after training. Johnson et al. [16] also reported that post-training scores increased by over 30% in some disciplines after training. Similarly, Inoue et al. [13] and Miring’u et al. [14] reported higher post-training scores while evaluating the NUITM-KEMRI biosafety training program and surveying knowledge and practices in the use of biosafety cabinets, respectively. Addo et al. [17] further observed that, in addition to increasing post-training scores, training also increased personnel motivation resulting in the strengthening of tuberculosis laboratory services in Ghana. The increase in scores usually suggests the extent of learning achieved. Modules can however be performed differently based on level of complexity and experience profiles of the trainees.

### Refresher and mentorship and training

Mentorship training is conducted following successful completion of classroom and practical sessions. It is also administered to previously trained laboratory staff upon a change in the scope of their work.

During mentorship training, the mentor works while the trainee observes or vice versa, depending on the prior experience of the trainee and complexity of the experiments being undertaken. An ideal mentor is usually a highly experienced BSL-3 laboratory staff who has had previous training and works in the laboratory on a regular basis. Mentorship allows trainees to practically apply knowledge and skills gained through the training and facilitates the development of expertise in a setting with increasingly minimized potential risks. Duration of mentored training depends on how fast a trainee demonstrates capability of working independently and the extent of his/her prior experience. Mentored training is monitored using a checklist that details core safety practices that must be learned.

Finally, refresher training is incorporated in the training program to sustain skills and for continuous learning. Previously trained users participate in all sessions of the training workshop apart from evaluations. Refresher training is also conducted when a new study is about to be started in order to update users on new standard operating procedures or safety strategies.

### Monthly biosafety meetings

Biosafety meetings are convened once per month for all trained BSL-3 laboratory staff. Accidents, incidents, or lessons learned are discussed, as well as ongoing activities, challenges being experienced while working in the laboratory, and other upcoming issues. Changes made to the operation protocols of each team are described where applicable. The biosafety officer further reports mechanical or operational faults if any, with safety precautions and course of action that the users can take in case of occurrence during a work session. Information sharing undertaken during such meetings provide a multi sectoral approach to training and awareness raising required by the interdisciplinary nature of life sciences.

## Discussion

**Fig. 1:**
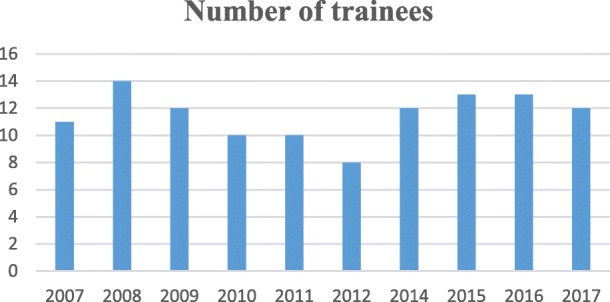
Number of trainees per annual biosafety training workshop

The training program is currently in its tenth year of implementation. One hundred fifteen laboratory staff have been trained so far as shown in Fig. 1 below.

A minimum number of trainees is enrolled each year. The small number allows for closer instruction especially during the hands-on training. Initially, trainees were predominantly junior researchers. The catchment has since expanded to include laboratory workers of diagnostic and reference laboratories. Observing that most of the trainees work in biosafety level two laboratories or enhanced level two laboratories, successful trainees who need to begin work in the BSL-3 laboratory are immediately enrolled in the mentorship program. Despite the limited number of active BSL-3 laboratories in the country, the demand for biosafety and biosecurity training is rising as more laboratories seek to standardize their operations towards quality accreditation alongside rising pressure for worker and public protection.

**Fig. 2:**
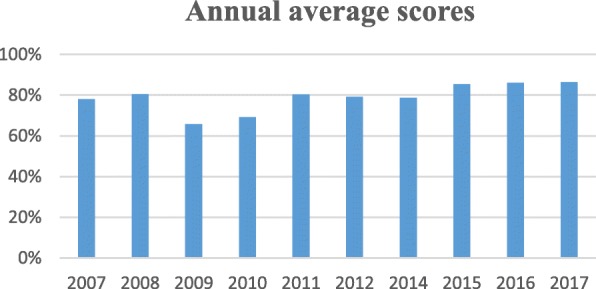
Trainee average scores in the annual biosafety level 3 training workshops

Figure 2 shows annual average scores since inception of the training program, computed from post-training written evaluations. The pass mark is usually 70%. Despite the absence of a clear trend, majority of the workshop scores have been above average. Further, modification of the program has been ongoing every year that has impacted the scores in one way or another. At inception, the biosafety training curriculum consisted of nine modules that majorly focused on functional and operational aspects of the BSL-3 laboratory and less on biosafety and biosecurity basic concepts and knowledge. Training content in this initial workshop was objectively focused on instilling skills and knowledge needed to work in a BSL-3 laboratory. The trainees were laboratory staff who were preparing to work in the BSL-3 laboratory, some of whom had access to the BSL-3 laboratory. Initial workshops also served the purpose of training of trainers, who would later be engaged in administering practical modules of the program.

In 2009, a review of the workshop’s outcomes in the first 2 years indicated the need to diversify training content in order to meet theoretical basic biosafety training needs. Training content was therefore revised to give more emphasis on core biosafety and biosecurity principles. Introductory modules were subsequently expanded to provide details on concepts such as the biocontainment principles, risk assessment of microorganisms, BSL-3 biosafety regulations, disinfection, and decontamination among others. Additional application modules were introduced to exemplify practical application of learned concepts for particular organisms. Further, the number of trainees per workshop was reduced to 12 to facilitate one-on-one practical training. The trainers provided questions for the written evaluation unlike in previous years whereby the organizers drafted the questions.

Further modification conducted prior to the 2010 workshop streamlined BSL-3 laboratory theoretical and practical training. Two introductory modules were replaced with application modules. A module on biosafety cabinets was added owing to the role they play in containing infectious agents coupled with the importance of correct usage for optimal containment. For sequential coverage of the concept of biocontainment, training content covering design and structural features of the BSL-3 was separated from the introductory module and merged with the maintenance of the BSL-3 laboratory module. To simplify practical training, a hands-on training checklist was extracted from the hands-on training theoretical module on practices in the BSL-3 laboratory. The structured checklist consists of a stepwise systematic procedure for working in the BSL-3 laboratory, starting with entry preparations done in the cell culture room to setting up and completing an experiment, and finally exit procedures. Further, trainee feedback forms were introduced to collect feedback from trainees on various aspects of the workshop for continuous improvement of the program. Previously trained biosafety staff were involved in delivering some of the lectures as well as in practical and discussion sessions.

Feedback from 2010 trainees indicated that the training content was too wide. In 2011, the curriculum was therefore reduced to 11 modules. Theoretical module on how to use the BSL-3 laboratory was combined with its practical session. Additionally, the BSL-3 laboratory by then had permanent staff who had completed mentorship training. Their participation in the hands-on training sessions positively influenced training outcomes due to increased contact between the trainers and the trainees. In 2012, a demonstration session on donning and doffing of PPE was added. Addition of this session helped to emphasize the role PPE plays in minimizing contact with biological materials as well as providing a rationale for correct donning and doffing of PPE at all biosafety levels. Additionally, the introductory module covering biosafety regulations was also revised to include biological and chemical waste management. Such wastes bear biological and chemical hazards respectively; hence, optimal caution in their handling is required.

In 2015, the workshop duration was extended to 3 days. The late morning and entire afternoon of the second day have since been allocated to practical training. The hands-on training is conducted through three or four groups, each with one or two trainers. Hands-on activity includes routine procedure for entry into the BSL-3 lab, specimen and equipment handling, and the exit procedure. Twelve modules were administered, including an additional application module covering level three bacterial agents. During the same year, a practical quiz was also included as part of the assessment to measure learning achieved during practical training. The quiz is administered immediately after the hands-on session.

As at 2017, need for training on practical execution of BSL-3 laboratory key routines emerged. Waste management, being a common routine was expanded to cover biological waste management and chemical waste management separately, since each type of waste bears different biosafety implications. Given the growing need for transportation of biological materials, necessitated by increasing popularity of multi-site studies, a module covering shipment of biological materials was added. Its content was based on international standards for packaging and shipment of biological materials. Biosafety level three bacterial agents application module, which previously covered biosafety and biosecurity of *Bacillus anthracis*, was also expanded to include a range of risk group three microorganisms with focus on pathogens that cause septicemia. To further improve basic biosafety and biosecurity awareness coupled with the importance of biosafety level two laboratories in Kenya, a module on working safely in BSL-2 laboratories was also included in the curriculum. Lastly, a module on biosecurity was added. This module describes biosecurity in the international context, stakeholders in biosecurity issues, biosecurity regulations in Kenya, ongoing biosecurity initiatives, and measures of improving biosecurity in research and diagnostic laboratories.

Currently, the curriculum consists of 13 modules including an introductory module, three modules on biosafety operations and regulations, three application modules, a module on biosafety in level two laboratories, four modules on BSL-3 laboratory, and a biosecurity module. Although the curriculum has not digressed from its initial objective, it has undergone several changes to effectively cover critical aspects of biosafety and biosecurity in the BSL-3 laboratory. An evaluation of the training program by Inoue et al. [13] indicated that it was able to significantly increase biosafety and biosecurity awareness post-training, with accompanying mentorship training and institutional support. The importance of practical training has also been acknowledged in the course of implementation of the program. Practical training through hands-on practice is widely recognized as means of instilling good biosafety and biosecurity practices among containment laboratories staff since it influences behavioral change that spills over to routine practices [18]. Practical training has therefore been extensively integrated into the training program, with almost half of the training duration dedicated for hands-on training. Trainee feedback on practical training has been positive all through.

## Conclusion

Implementation of the NUITM-KEMRI biosafety training program has been quite successful as confirmed by above average scores in the post-training evaluations as well as positive trainee feedback. Further, the training curriculum has been flexible enough to continuously accommodate emerging training needs, which has resulted to significant continuous improvement of the training program. A biosafety training program is therefore a critical tool in entrenching good biosafety and biosecurity in laboratory practices. Although many institutions have developed and administers laboratory biosafety training programs, peer-reviewed training programs are largely lacking. Many programs are implemented internally and maintained as internal institutional guidelines with minimal presence in peer-reviewed journals. Despite the differences in training content due to differing training needs and differences in settings, standardization of biosafety training as well as provision of reference for development or improvement of biosafety training programs is desirable.

## Abbreviations

BSL: Biosafety level
KEMRI: Kenya Medical Research Institute
NUITM: Nagasaki University Institute of Tropical Medicine
PPE: Personal Protective Equipment
WHO: World Health Organization
